# Depersonalization‐ and derealization‐like phenomena of epileptic origin

**DOI:** 10.1002/acn3.50870

**Published:** 2019-08-22

**Authors:** Lukas Heydrich, Guillaume Marillier, Nathan Evans, Margitta Seeck, Olaf Blanke

**Affiliations:** ^1^ Laboratory of Cognitive Neuroscience Brain‐Mind Institute, School of Life Sciences, Ecole Polytechnique Fédérale de Lausanne Lausanne Switzerland; ^2^ Department of Neurology University Hospital Geneva Geneva Switzerland; ^3^ Core Lab, Psychosomatic Competence Center, Department of Neurology Inselspital, Bern University Hospital, University of Bern Bern Switzerland; ^4^ Center for Neuroprosthetics, School of Life Sciences Ecole Polytechnique Fédérale de Lausanne Lausanne Switzerland

## Abstract

**Objective:**

Depersonalization refers to the sensation of being detached from one’s body, often associated with feelings of loss of control over one’s own body, actions, or thoughts. Derealization refers to the altered perception of one’s surroundings that is experienced as unreal. Although usually reported by psychiatric patients suffering from depression or anxiety, single case reports and small case series have described depersonalization‐ and derealization‐like symptoms in the context of epilepsy.

**Methods:**

We investigated the brain mechanisms of ictal depersonalization– and derealization like symptoms by analyzing clinical and neuropsychological data as well as the epileptogenic zone based on a multimodal approach in a group of patients reporting depersonalization‐ (*n* = 9) and derealization‐like symptoms (*n* = 7), from a single presurgical epilepsy center with focal epilepsy. We compared them with a group of control patients with experiential phenomena due to temporal lobe epilepsy (*n* = 28).

**Results:**

We show that all patients with ictal depersonalization‐like symptoms report altered self‐identification with their body and mostly suffer from frontal lobe epilepsy with the epileptogenic zone in the dorsal premotor cortex, while patients with derealization‐like symptoms suffer from temporal lobe epilepsy. This finding is supported by post‐ictal neuropsychological deficits, showing that depersonalization‐like symptoms were significantly more often associated with frontal lobe dysfunction as compared to the control patients and patients with derealization‐like symptoms.

**Conclusion:**

We argue that depersonalization of epileptic origin constitutes a distinct disorder due to frontal lobe epilepsy. We discuss these findings with respect to earlier accounts of depersonalization and the recent concept of bodily self‐consciousness.

## Introduction

During depersonalization (DP) patients report to be detached from and often associated with feelings of loss of control over one’s own body. These sensations may or may not be associated with an altered perception of one’s surroundings that is experienced as unreal, for example derealization (DR).^1^ Historically, it has been proposed that DP and DR constitute two distinct phenomena.^2^, ^3^, ^4^ However, currently depersonalization/derealization disorder (DDD) has been classified as a single dissociative disorder, requiring persistent and/or recurrent episodes of DP and/or DR.^5^

Although DP and DR are often reported by psychiatric patients suffering from depression^6^ and anxiety,^7^ and even though DSM‐V specifies that DDD should “not be attributable to another medical condition”, DP‐ and DR‐like phenomena have been reported in patients with epilpesy,^8^, ^9^, ^10^, ^11^, ^12^ after cortical electrical stimulation,^10^, ^11^ and in patients following traumatic brain injury.^8^, ^13^ However, these reports were in majority single case studies^12^ or small case series.^3^, ^8^, ^10^, ^11^ Moreover, due to the lack of studies applying quantitative lesion analysis, to date the phenomenological distinction between DP and DR as well as any potential distinct brain mechanisms remain poorly understood.^3^, ^14^

Sierra and Berrios^3^ highlight the role of the dorsolateral prefrontal cortex in DP, proposing that prefrontal hyperactivity and limbic inhibition results in a decrease of autonomic responses to emotional stimuli and thereby causing the sensation of detachment from the body and self.^15^, ^16^ DR on the other hand has been linked to the temporal–occipital cortex,^3^ in line with the observation of DR‐like experiences during experiential phenomena due to temporal lobe epilepsy (TLE).^4^, ^8^, ^9^ Simeon and colleagues^17^ have studied the brain mechanisms in a group of eight patients suffering from DDD using positron emission tomography (PET). They demonstrated a hypermetabolism in the parietal cortex, that correlated with symptoms of both persistent DP and DR, arguing that DP is due to a failure of integration of bodily signals in sensory association areas.

In the present study we aimed to investigate the neural correlates of DP and DR by analysing clinical and neuropsychological data in a group of patients with epilepsy suffering from focal epilepsy and ictal DP‐ and DR‐like phenomena. Given the historical link of both DP‐ and DR‐like phenomena as part of experiential phenomena with temporal lobe epilepsy,^4^, ^8^, ^9^ we also investigated a control group consisting of patients suffering from déjà vu or experiential hallucinations due to temporal lobe epilepsy, as reported previously.^18^ We localized the epileptogenic zone, using a multimodal approach as described previously.^18^, ^19^, ^20^, ^21^ In addition, we analyzed our patients’ symptoms with respect to the recently introduced concept of bodily self‐consciousness (BSC) (see 22). We hypothesized that the seizure onset, but also findings from the neuropsychological examination and the semiology in respect to alterations of BSC can be dissociated in patients suffering from DP‐like phenomena from patients with DR‐like phenomena and patients with other experiential phenomena.

## Material and Methods

### Patients

From a population of 450 patients suffering from intractable epilepsy undergoing presurgical evaluation in the Epilepsy Unit at the university hospital of Geneva between 1998 and 2011, we retrospectively selected patients meeting the following inclusion criteria: patients reporting (1) DP (e.g. illusory detachment from their body, as if being an outside observer, often associated with feelings of loss of control over one’s own body, actions, or thoughts) and (2) DR (e.g. altered perception of one’s surroundings that is experienced as strange or unreal) as part of their habitual seizure semiology*.* Moreover, patients reporting déjà vu (DV) or experiential hallucinations (EH) were selected from the same population as an additional control group (see 18). Patients were only included if they could be unequivocally assigned to one of the above mentioned four groups according to their semiology. Patients who suffered from DP and DR at the same time, were not included in the analysis. Selection of patients was based on the detailed clinical records taken at the time of evaluation. However, due to the retrospective nature of the study no systematic questionnaire could be employed. Patient reports were systematically analyzed for the three major aspects of BSC as reported previously, for example self‐identification with the body (i.e., the experience that the physical body and its parts belong to me), self‐location (i.e., the experience of where my body is located in space), and first–person perspective (i.e., the experience from where I experience to perceive the world).^22^, ^23^ Ethics approval was obtained from the Ethics committee at the university hospital of Geneva.

Basic patient characteristics (age, sex, handedness, seizure frequency, seizure duration, neurological examination, interictal und post‐ictal neuropsychological examination, psychiatric comorbidities, surgical therapy, postsurgical outcome) were compared over the four groups.

### Multimodal evaluation of the epileptogenic zone

All patients underwent phase I evaluation, including structural magnet resonance imaging (MRI) and functional imaging techniques [interictal and ictal surface electroencephalography (EEG), PET, ictal and interictal single photon emission computed tomography (SPECT)]. Eight patients underwent phase II evaluation, including intracranial EEG.

All imaging data were analyzed at the time of the original exploration and then reviewed for the purpose of this study by two of the authors in order to determine the epileptogenic zone (Lukas Heydrich and Guillaume Marillier). Anatomical structures were labeled according to the AAL atlas implemented in MRIcron (http://www.mccauslandcenter.sc.edu/mricro/mricron).

In order to illustrate the neural correlates underlying DP and DR we subsequently traced the epileptic zone for each patient on the T1 template using MRIcron.^19^, ^20^, ^24^ Structural lesions were identified using MRI. The functional relevance of these lesions was confirmed by a multimodality imaging approach,^25^, ^26^ which combines structural with coregistered functional imaging. This multimodal approach is classically used to improve the ability to detect and define the extent of temporal and extra‐temporal epileptogenic tissue.^26^ MRI brain scans were normalized to the smoothed T1 template using SPM5 (http://www.fil.ion.ucl.ac.uk/spm/software/spm5).^27^ As unified segmentation models give the most precise registration of lesioned structural images,^28^ no cost–function masking was necessary. Functional imaging (PET, SPECT) was normalized using SPM5 and coregistered to the normalized MRI scans. The epileptogenic tissue as suggested by the multimodality imaging was subsequently traced manually slice by slice either on the individual normalized brain scans or on the T1 weighted images using MRIcron (http://www.sph.sc.edu/comd/rorden/mricron).^24^ The later manual tracing on the template brain was only done when confidence could be achieved for matching corresponding slices between the lesioned brain and the template brain. If functional imaging highlighted brain areas adjacent to the structural lesions, these were included into the lesion analysis as well (following the approach used by 19). No patients with unclear lesion boundaries or metallic artifacts were included into the analysis. Lesion volumes (volume of interest, VOI) were determined as the sum of all voxels compromising the traced lesion in all slices and were spatially smoothed using a 5 mm full width at half maximum (FWHM) Gaussian Kernel and a threshold of 0.5.

The same procedure was applied to the control group (see 18).

### Statistical analysis

In a first step, the epileptic focus was attributed to the dominant or the nondominant hemisphere for language. Then, in a second step, patients were classified as suffering from seizures either due to temporal lobe epilepsy (TLE) and medial temporal lobe epilepsy (MTLE), respectively, frontal lobe epilepsy (FLE), parietal lobe epilepsy (PLE) or occipital lobe epilepsy (OLE).

For subsequent statistical analysis, we performed a chi‐square test for independent samples between the four groups, respectively the Fisher’s exact test, if the expected frequencies were <1.

Results of post‐ictal (in the immediate postictal period) and inter‐ictal neuropsychological evaluation including tests of executive function (word and figural fluency), verbal and visuo‐spatial memory, attention, gnosis, and language were also analyzed using a chi‐square test for independent samples, or the Fisher’s exact test, respectively, if the expected frequencies were <1. The *P*‐value was adjusted for multiple comparisons using the Holmes‐Bonferroni method.

## Results

### Description of the patient sample

#### Demographics and clinical characteristics

Sixteen patients undergoing presurgical evaluation in the Epilepsy unit at the university hospital of Geneva between 1998 and 2011 fulfilled the criteria for DP/DR and were selected retrospectively for this study: Nine patients with ictal DP and seven patients with ictal DR (for patients with DV and EH see below).

Demographic and clinical (see below) parameters did not significantly differ between the two groups (all *P* > 0.05, see Table 1). Six patients were male, 10 female, with an average age at evaluation of 33 years (SD 3.2 years). Fourteen patients were right‐handed (87.5%) and two were left‐handed (12.5%). The nondominant hemisphere for language was the right hemisphere in 15 cases (93%) and the left hemisphere in one case (7%), as confirmed using fMRI, WADA testing and/or neuropsychological assessment.

**Table 1:** Demographic patient characteristics in patients with DP, DR. DV and EH.

	Depersonalization *N* = 9	Derealization *N* = 7	Déjà vu *N* = 16	Experiential hallucinations *N* = 12	*P* value
Male/female	3/6	3/4	7/9	8/4	>0.05
Age at evaluation (years)	33.8	32.0	32.0	32.4	>0.05
Handedness (right/left/ambidextrous)	7/2/0	7/0/0	14/1/1	11/0/1	>0.05
Duration of epilepsy (years)	14.4	16.5	12.6	11.4	>0.05
Seizure frequency (p.a.)	335	562	238	153	>0.05
Neurological examination (normal/pathological)	4/5	4/3	12/4	9/3	>0.05
Family history (positive/negative)^a^	2/7	2/5	1/13	1/9	>0.05
Psychiatric diagnosis (yes/no)	0/9	1/6	3/5	4/2	0.019
Surgical therapy (yes/no)	7/2	5/2	13/3	9/3	>0.05
Favourable outcome after surgery (yes/no/unknown)^b^	4/3	4/1	13/0	6/2/1	0.05

a Information could not be retrieved retrospectively for all the patients, therefore *N* differs from the total *N*.

b Refers only to the patients being operated (*N* = 34). Significance level after correction for multiple comparisons (Bonferroni correction) = 0.004.

The average duration of epilepsy was 14 years (SD 12.9). Neurological examination was normal in eight (50%) patients. Three patients (out of 13 patients where a family history could be retrieved) presented with a positive family history for epilepsy (23 %). A psychiatric diagnosis prior to admission was present in two patients (12.5%, e.g. eating disorder and light to moderate depressive episode, panic disorder).

Surgical treatment was performed in 12 out of 16 patients (75%). Information about follow–up examinations was available in all patients after surgery. A favorable outcome (seizure free or significant seizure control after 3 months) was achieved in eight patients (66.6%). Four patients (33.3%) did not benefit from the surgical procedure.

For further details please refer to Table 1.

No significant difference regarding the demographic and clinical parameters was found between DP versus DR or versus the additional control group consisting of 28 patients reporting DV or EH (all *P* > 0.05).

For further details please refer to Table 1.

#### Semiology. Depersonalization

Nine patients reported a sensation of DP during their seizures. Seven patients reported the sensation of being detached from own bodily experience (e.g. touch), of whom four patients reported the sensation of full or hemi‐body numbness. One patient claimed that a stranger would have entered his body, while three patients said that they lost control over the body and their actions. Although no DP patient described a change in self‐location or first–person perspective, all DP patients reported disturbed self‐identification or body ownership with their body (to varying degrees).

Table 2 provides more detailed information on individual reports.

**Table 2:** Clinical characteristics and semiology in patients with DP.

Patient	Diagnosis	Lesion	Lesion analysis	Neurology	Semiology
DP 1	Epilepsy/dysplasia	Parietal cortex (L)	MRI, EEG, PET, SPECT	Vertigo and tinnitus	Feeling to lose the control over the right hemi‐body, feeling of the right arm being elevated while the right side of the trunk was lowered relative to the left side
DP 2	Epilepsy/oligodendroglioma	Frontal cortex (R)	MRI, EEG, PET, SPECT	Impaired short term and working memory	Altered touch (whole body), dissociation of body and mind (feeling detached of the body without leaving the body)
DP 3	Epilepsy/autoimmune	Temporal cortex (R)	MRI, EEG, PET, SPECT	Executive dysfunction	Feeling that someone enters her body, takes control of the body
DP 4	Epilepsy/dysplasia	Frontal cortex (R)	MRI, EEG, PET, SPECT	Normal	Feeling that his body is useless, is not feeling his body, he thinks that his body is disconnected from his head
DP 5	Epilepsy/DNET	Parieto‐occipital cortex (R)	MRI, EEG, PET, SPECT	Normal	Detachment of physical body, strong visual‐vestibular sensations
DP 6	Epilepsy/posttraumatic	Frontal and temporo‐parietal cortex (R)	MRI, EEG	Left hemi‐neglect (visual, sensory, auditory)/Anosognosia and prosopagnosia	Detachment of physical body, strong visual‐vestibular sensations
DP 7	Epilepsy/inflammatory lesion	Frontal cortex (L)	MRI, EEG, SPECT	Discrete motor hemi‐syndrome right/Semantic paraphasia	Altered touch of the right hand has changed, right side of body feels strange
DP 8	Epilepsy/neurocysteriosis	Frontal cortex, Insula (R)	MRI, EEG	Left hemi‐spatial neglect/left‐sided diadochokinesis and dysmetria	Loosing control of left hand, detachment and feeling of a presence
DP 9	Epilepsy/vasculitis	Frontal and occipital cortex (L)	MRI, EEG	Right sided sensorimotor hemi‐syndrome, right hemianopia	Sensation of body distortion, detachment

PET, positron emission tomography; MRI, magnet resonance imaging; EEG, electroencephalography; SPECT, single photon emission computed tomography; MNI, Montreal Neurological Institute.

#### Semiology. Derealization

DR was reported by seven patients and mostly involved the sensation as if the environment felt unreal, far away, perceived through a veil, like in a dream or being in a film. No change of self‐location, first–person perspective or self‐identification was reported by any of the patients with DR.

### Neuropsychological testing

Interictal neuropsychological testing was available in 15 out of the 16 patients (93%) suffering from DP or DR. Post‐ictal neuropsychological testing was available in nine patients (56%). Testing of frontal lobe functions (e.g. executive functions, such as fluency) in the immediate postictal phase was significantly more often pathologic in patients reporting DP (78%) as compared to DR (0%; Chi^2^ = 6.78, *P* < 0.01), as well as DV and EH (Chi^2^ = 20.3, *P* < 0.001). None of the other neuropsychological test scores (inter‐ and post‐ictal) showed a significant difference between DP and DR.

Comparing DP, DR, EH and DV showed a significant effect of post‐ictal (*P* = 0.012) and inter‐ictal (*P* = 0.038) language problems being more frequent in the group of patients suffering from EH as compared to DP, DR and DV.

### The epileptogenic zone based on a multimodal evaluation

Surface EEG was available in 100% of the patients, intracranial EEG was available in 18% of the patients. MRI in 100% of the patients, volumetry in 25% of the patients, spectroscopy in 43% of the patients, PET in 68% of the patients, interictal SPECT in 68% of the patients, and ictal SPECT in 62.5% of the patients. No statistical difference for the availability of the different imaging techniques was found between any of the groups (all *P* > 0.05).

DP (*N* = 9) was associated with a seizure onset in nondominant hemisphere for seven patients (77%) and in the dominant hemisphere in two patients (23%). DR (*N* = 7) was associated with a seizure onset on the nondominant hemisphere in four patients (57%) and the dominant hemisphere in three patients (43%, n.s.). In seven patients with DP a multimodal evaluation identified the frontal lobe as the primary epileptic focus (*n* = 7; 77%), whereas none of the patients suffering from DR had frontal lobe involvement (e.g. FLE; Chi^2^ = 6.78, *P* < 0.01). Two patients with DP (23%) and six patients with DR (85%) were primarily suffering from MTLE (Chi^2^ = 6.34, *P* = 0.01). We then compared the seizure onset zone in patients suffering from DP and DR with the seizure onset zone in patients suffering from DV and EH due to MTLE. Statistical analysis revealed that patients with DP were suffering significantly more often from frontal lobe epilepsy as compared to DV, DR, EH (*x* ^2^ = 20.3, *P* < 0.001), while DR, DV and EH could be linked to MTLE as compared to DP (*x* ^2^ = 12.96, *P* < 0.001). The dominant hemisphere for language was significantly more often involved in patients with EH as compared to the other three other groups (DV, DR, DP), which were mostly due to a seizure onset zone in the nondominant hemisphere for language (*x* ^2^ = 10.13, *P* = 0.02).

### Maximal overlap of the epileptogenic zone

**Figure 1:**
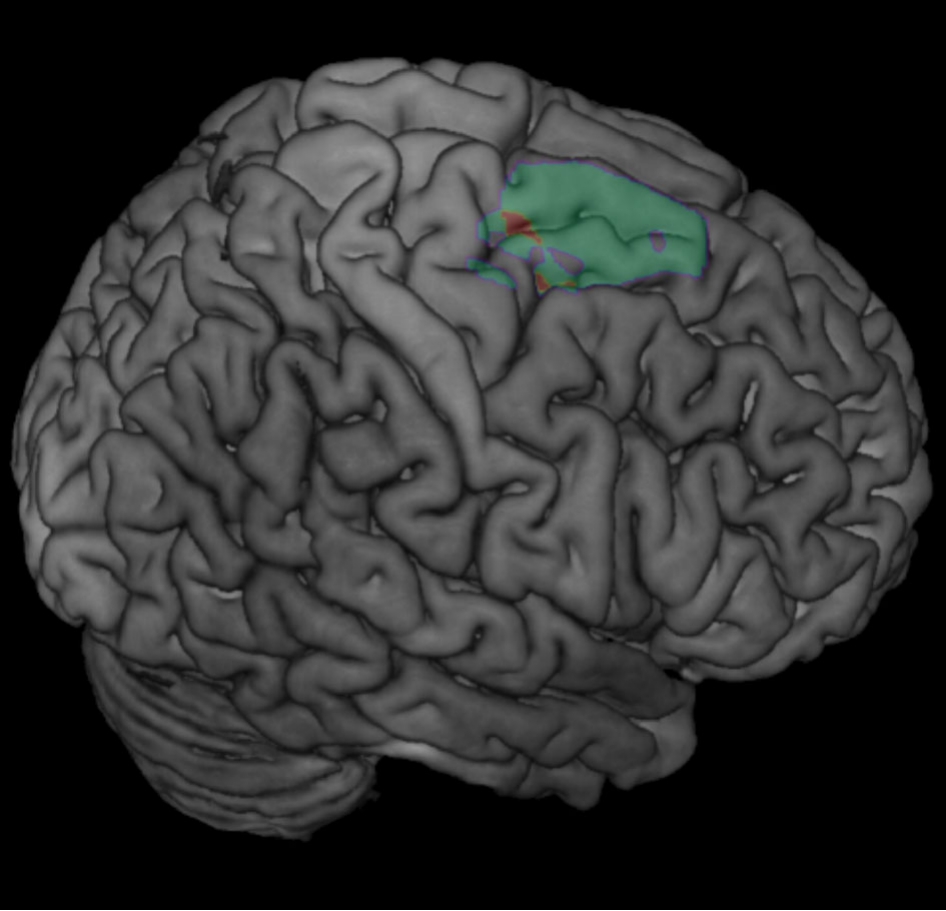
Epileptogenic zone in patients with ictal depersonalization‐like phenomena. Lesion overlap analysis highlighted the right medio‐dorsal premotor cortex (PMC) [cantered on Montreal Neurological Institute (MNI) *x* = 18, *y* = −5, *z* = 59, Brodmann area 6], which was found to be involved in in five out of nine patients with DP. The number of overlapping lesions is illustrated by color, from violet (*n* = 3) to red (maximal lesion overlap, *n* = 5).

**Figure 2:**
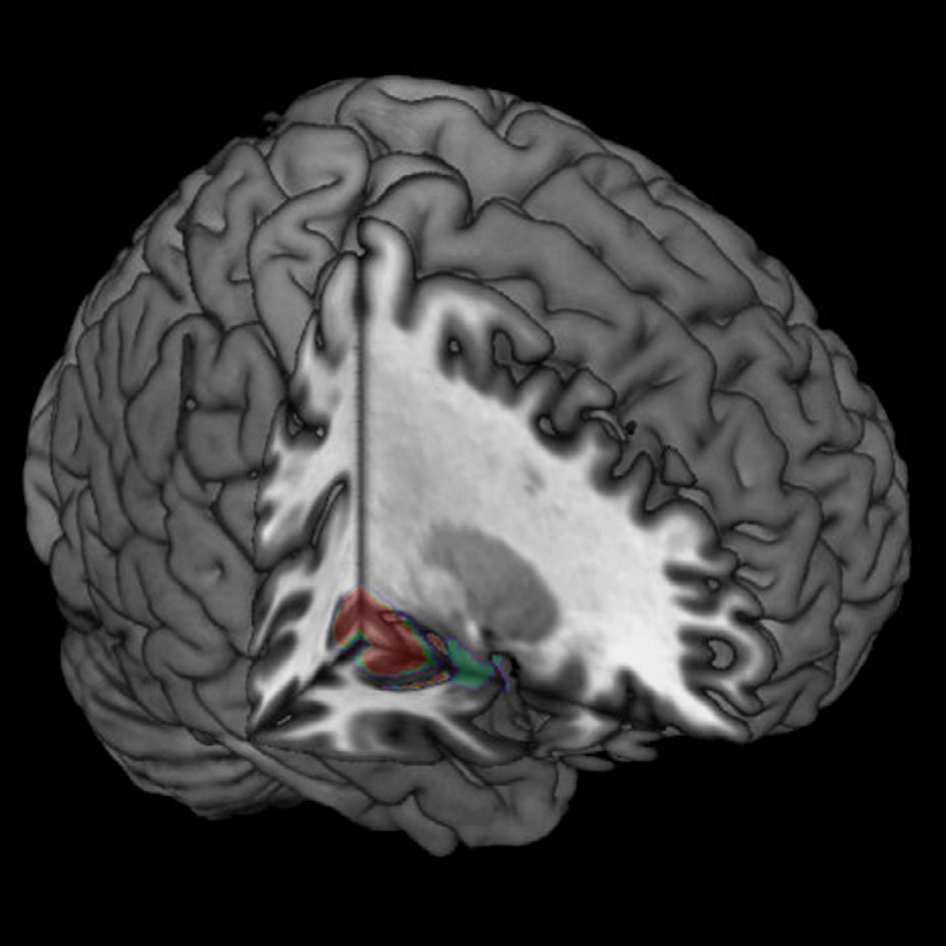
Epileptogenic zone in patients with ictal derealization‐like phenomena. Lesion overlap analysis highlighted the right posterior mesial temporal lobe (MTL) [centered on Montreal Neurological Institute (MNI) *x* = 28, *y* = −21, *z* = −14], which was found to be involved in four out of seven patients with DR. The number of overlapping lesions is illustrated by color, from violet (*n* = 2) to red (maximal lesion overlap, *n* = 4).

Figures 1 and 2 shows the results of the voxel–based overlap analysis of the epileptogenic zone. In the DP group, our analysis revealed the right medio‐dorsal premotor cortex (PMC) as the region of maximal overlap. This zone was anterior to the precentral gyrus and mainly involved the superior frontal gyrus, extending towards the supplementary motor area and the medial prefrontal cortex. Maximal overlap was found for five out of nine patients [Montreal Neurological Institute (MNI) *x* = 18, *y* = −5, *z* = 59, Brodman area 6, Fig. 1]. For the DR group the same analysis highlighted the right MTL (maximal overlap in the right hippocampus/posterior MTL in four out of seven patients, MNI *x* = 28, *y* = −21, *z* = −14, Fig. 2).

For the display of the seizure onset zone in patients with DV and EH please refer to the original work by Heydrich et al.^18^

## Discussion

In the present study we investigated the brain mechanisms of DP and compared them with those associated with DR in a group of patients suffering from pharmaco–resistant epilepsy. Overall, DP was rare and only found in approximately 3.5% in our sample. All DP patients reported altered self‐identification with their body during their seizures (and normal self‐location and first–person perspective), while no change in BSC was reported in DR. DP was due to FLE in seven out of nine patients, while DR (as well as DV and EH, which served as a control group), were linked to MTLE. Using **a** multimodal approach we were able to demonstrate that the epileptogenic zone in patients with DP was located in the medio‐dorsal premotor cortex, extending towards the supplementary motor area and the medial prefrontal cortex. This finding was also supported by the analysis of the post‐ictal neuropsychological exam showing that DP was significantly more often associated with a (pre‐) frontal lobe dysfunction, for example executive dysfunction, as compared to DR, DV and EH.

To the best of our knowledge there are only anecdotal data on the neuropsychological profile in patients suffering from DP.^8^ Interestingly, Guralnik et al.^29^ demonstrated in a group of 21 patients suffering from DDD a disruption in early perceptual and attentional processes, reflecting a dysfunction of a dorsal fronto‐parietal network^30^ and thus supporting our data to a certain extent. However, we note the limitation of analysing the results of the immediate post‐ictal neuropsychological examination, for example neuropsychological examination not being possible or incomplete in half of the patients. We also not that a direct comparison of the neuropsychological profile of patients suffering from persistent DP and those suffering of DP of epileptic origin is difficult. Therefore, a more systematic, ideally prospective approach is needed to better establish the neuropsychological profile in patients suffering from DP of neurological origin.

Ictal DP was characterized by the illusory perception of being detached from one’s own body experience (e.g. sensation of full or hemi‐body numbness) and emotions, to no longer identify with the body, including the sensation of losing control over the body. This resembles what has been described in patients suffering from DP in the context of psychiatric disorders, including DDD,^8^, ^31^ although the latter requires persistent and/or recurrent episodes of depersonalization and/or derealization^5^ and it has been proven difficult to distinguish DP due to an underlying organic disease from DP in the context of a (coexisting) psychiatric disorder.^8^

Importantly, based on subjective reports, DP could be dissociated from DR in our patient sample. Patients suffering from DR did not describe a detachment from the body but rather from the nonbodily surroundings, for example that things seemed unreal, far away or even distorted and like in a dream. Finally, concerning the three key aspects of BSC, only self‐identification with the body or body ownership was abnormal in patients with DP, whereas self‐location and first–person perspective were unaffected. This differed from patients with DR‐like symptoms, in whom no alterations of BSC were noted.

These subjective reports illustrate that DP can not only be differentiated from DR, but also from other complex illusory own body perceptions of neurological origin, especially autoscopic phenomena, such as the feeling of a presence,^21^ out of body experiences (OBE)^19^ and heautoscopy (HAS).^20^, ^23^ While in the latter two conditions patients might also report a certain degree of depersonalization,^20^ it should be noted that in addition to the sensation of self‐detachment and loss of self‐identification with the physical body, a strong identification with an additional illusory own body (visual or nonvisual, for example HAS, OBE) and/or a change of self‐location and change of the first–person perspective (e.g. OBE) is required in order to fulfil the diagnostic criteria for autoscopic phenomena.^22^ Although patients with DP experience the body as foreign, numb and not belonging to oneself or may describe the mental state characterizing DP as if they were looking at themselves from the outside, none of our patients with ictal DP reported an actual change of the first–person perspective. Thus, compared to patients with OBEs, DP patients usually state that it is a feeling “as if” they were outside observers of themselves, more comparable to a belief than an illusory visual perception. However, more detailed studies are required to investigate the presence of autoscopic phenomena and all three key aspects of BSC in patients with DP.

How do these data in patients with DP, relate to recent data on the neural correlates of bodily self‐consciousness, involving self‐identification or ownership for the body? Based on clinical, behavioral and neuroimaging data, it has been suggested that multisensory integration in the premotor cortex is a key mechanisms for hand ownership and self‐identification for the full body.^32^, ^33^, ^34^ Moreover, the dorsal and ventral PMC is a region well‐known for its importance in the integration of sensorimotor and multisensory bodily signals.^35^ PMC processes visual, tactile and sensory information, related to body parts,^33^, ^35^, ^36^ the whole body^37^ or signals across different body parts.^32^ Thus, perceived self‐identification with a full body has been linked to and positively correlated with activity of bilateral PMC.^32^ Thus, we argue that aberrant epileptic activity in the PMC results in a loss of self‐identification with body parts of the full body (for discussion see also 12) due to abnormal processing in multisensory regions, while self‐location and first–person perspective, which have been linked to more posterior brain regions (centering at the temporo‐parietal junction and the posterior insula)^19^, ^20^, ^38^ remain relatively unaffected.

A careful review of the individual reports given by patients with DP reveals not only a detachment from bodily experience (e.g. loss of self‐identification), but also a diminished sense of control over one’s actions, for example a loss of their sense of agency for their body and movements (e.g. patient 4 reported to feel like a robot without control over his actions and as if losing his identity). We note that the zone of maximal overlap of the epileptic focus in the dorsal PMC extended medially to include the supplementary motor area and the medial prefrontal cortex that have both been involved in the sense of agency and self‐identification.^39^, ^40^, ^41^ The supplementary motor area and the dorsal premotor cortex are well known for their importance for motor control, motor awareness and the sense of agency.^40^ Thus, electric cortical stimulation of the premotor cortex results in involuntary movements without awareness^42^, ^43^ and epileptic seizures originating in the supplementary motor area and the medial prefrontal cortex can cause ictal alien hand phenomena.^44^ Moreover, the medial prefrontal cortex has been linked to cognitive aspects of self–related processing,^45^ such as self‐reference, self‐concept and a mental representation of oneself as a subject of experience.^46^ Thus, epileptic activity in the dorsal premotor cortex and propagation to adjacent supplementary motor cortex and medial prefrontal cortex might also interfere with agentive and cognitive aspects of self‐consciousness, as seen in DP. Our data are in line with the account of depersonalization being a disorder hypoemotionality due to a corticolimbic disconnection put forward by Sierra and colleagues.^47^, ^48^ While Sierra and Berrios suggest a link between dorsolateral prefrontal hyperactivity and limbic inhibition (e.g. in the anterior cingulate cortex, amygdala, insula), resulting in a decrease of autonomic response to emotional stimuli,^15^, ^16^ the present data are in line with the role of the dorsal medial prefrontal cortex in emotion regulation without direct involvement of limbic structures.^49^, ^50^

We note that our finding of the epileptogenic zone being located in the PMC is in contrast with the study of Simeon et al.^17^ showing that hypermetabolism in the parietal cortex in eight patients suffering from DDD is positively correlated with depersonalization scores. The parietal cortex has also been linked to other conditions with illusory own body perception, such as the alien limb phenomenon^51^ or postural phantom limb sensation.^52^ However, this might be because patients with DDD unlike our patients report both symptoms of DP and DR at the same and that it is difficult to directly compare patients with DDD and patients suffering from DP‐like symptoms of epileptic origin. Also, we argue that DP due to epileptic activity in the PMC is associated with a loss of function (loss of self‐identification, loss of agency), which would be rather reflected by a PET hypometabolism instead of a hypermetabolism.

A limitation of the study is the fact that, despite the extensive presurgical workup, in only 66% of our patients a favorable outcome could be achieved and that only a 3‐month follow‐up was retained in all patients. However, we note that the percentage of favorable outcome is in line with the numbers reported in the literature and that the majority of recurrence of seizures (e.g. 80%) are observed within the first 6 months after the surgical procedure.^53^, ^54^

In conclusion, we demonstrate for the first time that DP of epileptic origin can be linked to the medio‐dorsal PMC. We show that by applying a structured comprehensive analysis of semiology, detailed neuropsychological evaluation, and by using quantitative lesion analysis, DP and DR of epileptic origin can be dissociated, for example that DR (together with other experiential phenomena, such as DV and EH) is part of the semiology observed in patients suffering from MTLE while DP can be linked to FLE and a seizure onset zone in PMC. This supports the role of the PMC as a part of a neural network underlying bodily self–consciousness (e.g. self‐identification) due to multisensory integration.

## Author Contributions

Lukas Heydrich designed the study, collected and analyzed the data and wrote the manuscript.

Guillaume Marillier collected the data.

Nathan Evans analyzed the data and helped writing the manuscript.

Margitta Seeck helped collecting the data and writing the manuscript.

Olaf Blanke supervised the project and helped writing the manuscript.

## Conflict of Interest

The authors report no conflict of interest and no financial relationships relevant to the manuscript.
